# Norepinephrine Modulates Pyramidal Cell Synaptic Properties in the Anterior Piriform Cortex of Mice: Age-Dependent Effects of β-adrenoceptors

**DOI:** 10.3389/fncel.2015.00450

**Published:** 2015-11-19

**Authors:** Abhinaba Ghosh, Nicole C. Purchase, Xihua Chen, Qi Yuan

**Affiliations:** Division of BioMedical Sciences, Faculty of Medicine, Memorial University of Newfoundland, St. John’sNL, Canada

**Keywords:** norepinephrine, β-adrenoceptor, pyriform cortex, early odor preference learning, miniature EPSC, miniature IPSC

## Abstract

Early odor preference learning in rodents occurs within a sensitive period [≤postnatal day (P)10–12], during which pups show a heightened ability to form an odor preference when a novel odor is paired with a tactile stimulation (e.g., stroking). Norepinephrine (NE) release from the locus coeruleus during stroking mediates this learning. However, in older pups, stroking loses its ability to induce learning. The cellular and circuitry mechanisms underpinning the sensitive period for odor preference learning is not well understood. We first established the sensitive period learning model in mice – odor paired with stroking induced odor preference in P8 but not P14 mice. This learning was dependent on NE-β-adrenoceptors as it was prevented by propranolol injection prior to training. We then tested whether there are developmental changes in pyramidal cell excitability and NE responsiveness in the anterior piriform cortex (aPC) in mouse pups. Although significant differences of pyramidal cell intrinsic properties were found in two age groups (P8–11 and P14+), NE at two concentrations (0.1 and 10 μM) did not alter intrinsic properties in either group. In contrast, in P8–11 pups, NE at 0.1 μM presynaptically decreased miniature IPSC and increased miniature EPSC frequencies. These effects were reversed with a higher dose of NE (10 μM), suggesting involvement of different adrenoceptor subtypes. In P14+ pups, NE at higher doses (1 and 10 μM) acted both pre- and postsynaptically to promote inhibition. These results suggest that enhanced synaptic excitation and reduced inhibition by NE in the aPC network may underlie the sensitive period.

## Introduction

Critical periods of early postnatal life for neural circuitry development and plasticity occur in several sensory systems (Hensch, 2004). In olfaction, early odor preference learning as a form of classical conditioning has been extensively studied. Neonatal rodents can be conditioned to prefer novel odors (conditioned stimulus, CS) that are paired with various stimuli mimicking maternal care or even maltreatment (unconditioned stimulus, UCS), such as stroking/tactile stimulation (Sullivan and Leon, 1986; McLean et al., 1993; Morrison et al., 2013; Roth et al., 2013), milk (Johanson and Hall, 1979, 1982), and mild shock (Camp and Rudy, 1988; Roth and Sullivan, 2001; Moriceau et al., 2006; Roth et al., 2013). Early odor preference learning can have life-long influences, for example, affecting sexual preference in adult rats (Fillion and Blass, 1986). However, such preference learning is only acquired during the first one and a half weeks of life; beyond this period, stroking loses its effectiveness as a UCS (Woo and Leon, 1987) and mild shock induces odor aversion instead of preference (Camp and Rudy, 1988; Sullivan et al., 2000a; Roth et al., 2013).

Several lines of evidence have suggested that neuromodulator norepinephrine (NE) is critical in early odor preference learning and it serves as a UCS signal in this model. NE is released from the locus coeruleus (LC) following tactile stimulation (Nakamura et al., 1987; Nakamura and Sakaguchi, 1990; Rangel and Leon, 1995) and pharmacological blocking of either α- or β-adrenoceptors in the olfactory bulb (OB; Sullivan et al., 2000b; Shakhawat et al., 2012), or the anterior piriform cortex (aPC; Morrison et al., 2013), prevents early odor preference learning in rat pups. Additionally, odor preference learning can be acquired when a novel odor is paired with α- or β-adrenoceptor activation (Sullivan et al., 2000b; Harley et al., 2006; Morrison et al., 2013).

The cellular effects of α- and β-adrenoceptor activation that may underlie odor learning have been characterized in both the OB and aPC. NE disinhibits mitral cells from granule cells via α_2_-adrenoceptors (Trombley, 1994; Pandipati et al., 2010) and results in long-term enhancement of gamma oscillations in the OB (Pandipati et al., 2010). β-adrenoceptor activation has also been shown to promote mitral cell long-term potentiation (LTP)-like plasticity *via* disinhibition of mitral cells in the OB (Yuan, 2009), and to enhance pyramidal cell LTP and short-term plasticity in the aPC (Best and Wilson, 2004; Morrison et al., 2013). The acute effects of β-adrenoceptor during odor conditioning are hypothesized to promote NMDA receptor (NMDAR)-mediated associative LTP and learning. Blocking NMDA receptors, either systemically (Lincoln et al., 1988; Weldon et al., 1997), or locally in the OB (Lethbridge et al., 2012) or aPC (Morrison et al., 2013), prevents early odor preference learning in rat pups.

What determines the critical window for early odor preference learning and its termination? It has been suggested that the sensitive period for the tactile stimulation to induce learning is related to the development of α_2_-adrenoceptor autoinhibition in the LC, which appears to alter LC firing patterns with age (Kimura and Nakamura, 1987; Nakamura et al., 1987). However, recent evidence also shows strong age-dependent effects of NE in the OB. Diminished NE induced-gamma oscillation correlates with reduced α_2_-adrenoceptor mediated disinhibition in older animals (Pandipati and Schoppa, 2012). Developmental downregulation of NMDAR (Franks and Isaacson, 2006) and reduced NMDAR-dependent LTP at the mitral cell to pyramidal cell synapses in the aPC of >P10 pups (Poo and Isaacson, 2007) provide additional support for a critical period window. Whether NE and adrenoceptors demonstrate age-dependent changes in the aPC has not been shown.

Here we first replicated the sensitive period odor learning model in mice (Roth et al., 2013) and established the role of β-adrenoceptors in learning. We then characterized the dose-dependent effects of NE on the intrinsic and synaptic properties of aPC pyramidal cells. We found that β-adrenoceptors mediate NE modulation of miniature EPSCs (mEPSCs) and miniature IPSCs (mIPSCs) in brain slices of P8–11 pups.

## Materials and Methods

### Experimental Subjects and Ethics Statement

C57B1/6J mouse pups (Charles River, Canada) of either sex were used in this study. All animals were bred in Memorial University of Newfoundland’s Animal Care Facility. Dams were provided with *ad libitum* access to food and water. Day of parturition was considered 0 days of age; i.e., P0. Subjects aged P8–11 or P14–21 (P14+) were used for experimentation. Pups were arbitrarily selected and assigned to training conditions from each litter. Conditioning and testing were performed in a temperature-controlled room (∼28°C). All experimental procedures were approved by the Institutional Animal Care Committee at the Memorial University of Newfoundland in accordance with the Canadian Council on Animal Care guidelines.

### Odor Preference Training and Testing

All pups were trained and tested individually for behavioral experiments. Following a 10 min habituation in a training box with unscented corn-cob bedding, pups were placed in a training box with peppermint-scented corn cob bedding (0.3 mL of odorant extract in 500 mL of bedding). Subjects in the odor preference training group (O/S^+^) were stroked on the back with a paintbrush for 30 s, followed by a 30 s rest interval, repeating for a total of 10 min. Subjects in the control group (odor only, O/S^-^) were placed on the peppermint-scented corn cob bedding (without stroking) for 10 min. Pups were returned to home cage until testing.

Twenty-four hours after training, pups were individually tested for odor preference. The order of pups tested with respect to their experimental condition was random. The testing apparatus was a stainless steel box (30 cm × 20 cm × 18 cm) placed over two training boxes with a mesh floor. One box contained peppermint-scented corn cob bedding and the other contained unscented bedding. There was a 2 cm neutral zone in the center of the apparatus separating the two boxes. Each test involved placing a pup in the neutral zone, and recording the time spent over each box (peppermint vs. unscented). This was recorded for five trials of 60 s each, with a 60 s interval between trials. Starting orientation of the pup was counterbalanced over the trials. The testing apparatus was cleaned with 70% ethanol after each trial. The percentage of time spent over the peppermint bedding with respect to the total time spent in both beddings was calculated.

#### Propranolol Injection

Mouse pups (P8) were randomly assigned to experimental groups and weighed. Pups were injected with 25 μl of either saline or propranolol (20 mg/kg, s.c.). Pups were returned to the home cage for 30 min before habituation and training.

### *Ex Vivo* Electrophysiology

Naive pups from both age groups were anesthetized in a glass jar with pure isoflurane drops followed by decapitation. Brains were quickly removed and placed in cold (2–4°C) sucrose based solution containing: (in mM) 83 NaCl, 2.5 KCl, 3.3 MgSO_4_, 1 NaH_2_PO_4_, 26.2 NaHCO_3_, 22 glucose, 72 sucrose, 0.5 CaCl_2_, bubbled with 95% O_2_ & 5% CO_2_ (Morrison et al., 2013). Para-sagittal slices (300 μm) were cut in a vibratome (Leica) and incubated in the same sucrose based solution for 30 min at 35°C before the experiment.

An open bath recording chamber was continuously perfused with warm (30–32°C) artificial CSF (aCSF) containing (in mM): 110 NaCl, 2.5 KCl, 1.3 MgSO_4_, 1 NaH_2_PO_4_, 26.2 NaHCO_3_, 22 glucose, 2.5 CaCl_2,_ at the rate of 2–3 mL/min. For EPSC recording, aCSF containing (in mM) 119 NaCl, 5 KCl, 4 CaCl_2_, 4 MgSO_4_, 1 NaH_2_PO_4_, 26.2 NaHCO_3_, 22 glucose was used (Poo and Isaacson, 2007). Olympus BX51WI upright microscope was used for viewing the slices in differential interference contrast with 40× magnification.

Recordings of layer II pyramidal cells in the aPC were performed with glass micropipettes (resistance 3–6 MΩ) pulled by a Flaming/Brown micropipette puller (P-97, Stutter Instrument Co., USA) and filled with intrapipette solution containing (in mM): 123 K-gluconate; 2 MgCl_2_; 8 KCl; 0.2 EGTA; 10 HEPES; 4 Na_2_-ATP; 0.3 Na-GTP for recording intrinsic properties and miniature EPSC. For IPSC experiments, micropipettes were filled with intrapipette solution containing (in mM): 123 KCl; 2 MgCl_2_; 8 KCl; 0.2 EGTA; 10 HEPES; 4 Na_2_-ATP; 0.3 Na-GTP; thus chloride reversal potential being 0 mV. Cells with access resistance >20 MΩ or change more than 30% during the recording were excluded from mEPSC and mIPSC analysis.

For measuring intrinsic properties, depolarization currents of increasing amplitude (10 pA steps) were injected into the cell through the patch-pipette in current clamp mode. The action potential (AP) evoked by the smallest current injection was used for measuring threshold, amplitude, half width, rise time and decay time. Input resistance was measured by taking average of multiple (5 or more) traces following 10 pA hyperpolarizing current injection.

For synaptic properties, cells were held at –70 mV in voltage-clamp mode. Miniature IPSC (mIPSC) was measured in the presence of tetrodotoxin (0.5 μM; Tocris), D-APV (50 μM; Tocris) and NBQX (40 μM; Tocris) in aCSF. Miniature EPSC (mEPSC) was measured in the presence of tetrodotoxin (0.5 μM) and picrotoxin (100 μM; Tocris). Cells were recorded for 5 min in each NE dose (0.1, 1 and 10 μM; NE bitartrate salt monohydrate, Sigma–Aldrich) in an accumulative manner. β-adrenoceptor antagonist propranolol hydrochloride (20 μM: Sigma–Aldrich) and α-adrenoceptor antagonist phentolamine hydrochloride (50 μM; Sigma–Aldrich) were added to the aCSF to test the specificity of NE at various doses on adrenoceptors. Antagonists were washed in for >10 min before adding NE. The specific effects of β-adrenoceptors were tested with propranolol in the presence of NE.

Multiclamp 700B amplifier and pClamp10 software were used for data acquisition (filtered with 2 kHz low pass filter) and digitization (10 kHz sampling frequency).

Electrophysioloigical data were analyzed by Clampfit and Igor. For miniature IPSC and EPSC, Mini Analysis Program was used to analyze the frequency and amplitude of the events.

### *Post Hoc* Histology

In a subset of recorded cells, biocytin (0.1%) was added to the intrapipette solution and the recorded cell was filled up during the experiment. Slices were transferred to 4% paraformaldehyde solution, left at 4°C until further processing. Slices were washed with phosphate buffer saline (PBS) afterward. Then the slices were incubated with CY3-conjugated Streptavidin (1:1000 in PBS) at room temperature for 2 h, washed with PBS, mounted onto slides, and imaged at 40× magnification with an Olympus Fluoview FV1000 confocal microscope.

### Statistics

OriginPro 9.0 was used to analyze all datasets. Data were reported as mean ± SEM. One way ANOVA and Student’s *t*-test (two-sample, two-tailed) were used for behavioral studies and comparisons of intrinsic electrophysiological properties. Paired two-sample, two-tailed *t*-tests were used for all experiments characterizing dose-dependent NE synaptic effects.

## Results

### Sensitive Period Early Odor Preference Learning in Mice

**FIGURE 1:**
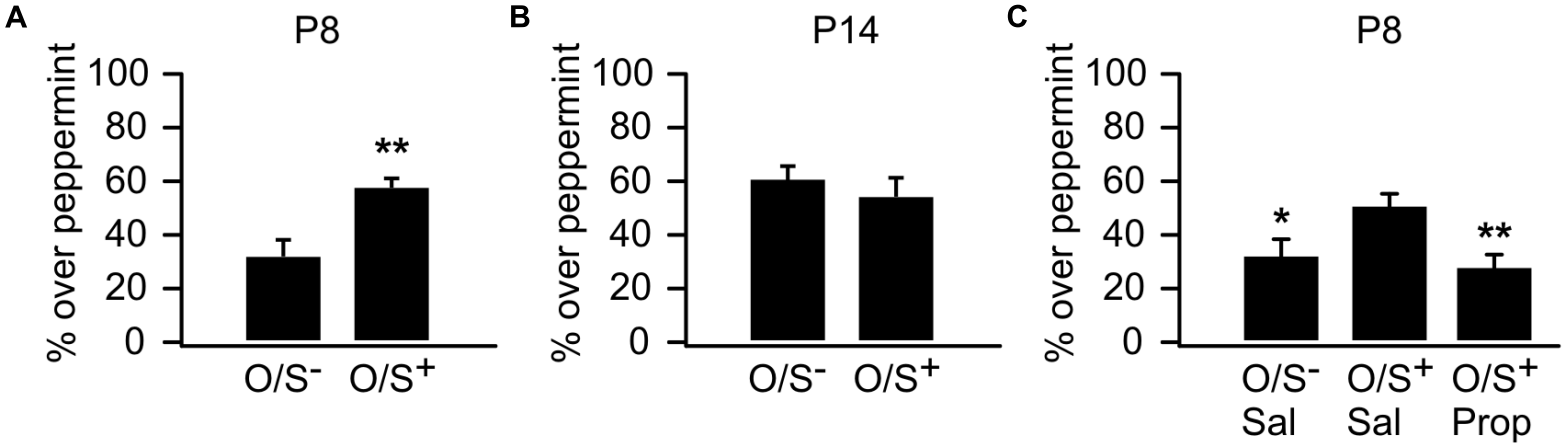
**Odor preference learning in mice.** **(A)** Percentage of time spent over peppermint-scented bedding in P8 pups. **(B)** Percentage of time spent over peppermint-scented bedding in P14 pups. **(C)** Percentage of time spent over peppermint-scented bedding in P8 animals with either propranolol or saline injections. O/S^+^: odor paired with stroking. O/S^-^: odor only without stroking. ^∗^*p* < 0.05; ^∗∗^*p* < 0.01.

We first replicated sensitive period odor learning in mice, which was well established in rat pups (Wilson and Sullivan, 1994; Yuan et al., 2014) and recently in mice (Roth et al., 2013). P8 mouse pups in the O/S^+^ group spent significantly more time in the peppermint bedding (57.25 ± 3.92%, *n* = 10) than the O/S^-^ group (32.56 ± 5.14%, *n* = 10, *t* = 3.82, *p* = 0.001; **Figure 1A**). However, P14 pups with O/S^+^ training (52.86 ± 7.95%, *n* = 6) did not show a significant difference from those in the O/S^-^ group (61 ± 4.55%, *n* = 8, *t* = 0.94, *p* = 0.363; **Figure 1B**). Interestingly, mouse pups at P14 lost the mild aversion to peppermint which was evident in P8 pups.

We next tested whether the early odor preference learning in P8 mice was dependent on NE β-adrenoceptors as in rat pups (Sullivan et al., 2000b). When β-adrenoceptor blocker propranolol was injected before training, mouse pups failed to form an odor preference compared to saline O/S^+^ controls. A one-way ANOVA reveals a significant difference among groups (*F*_2,39_ = 5.73, *p* = 0.006; **Figure 1C**). *Post hoc* Fisher test demonstrated significant differences between the propranolol injected O/S^+^ group (28.13 ± 5.18%, *n* = 15) and the saline O/S^+^ group (50.71 ± 4.97%, *n* = 18, *t* = 3.18, *p* = 0.002), while the propranolol group was not different from the saline O/S^-^ group (31.64 ± 6.32%, *n* = 9, *t* = 0.40, *p* = 0.684). These results suggest early odor preference learning in mouse pups is dependent on NE release acting *via* β-adrenoceptors.

### Effects of NE on Pyramidal Cells in the Piriform Cortex

To understand the role of NE in the piriform cortex that may underlie early odor preference learning, we then compared the effects of NE on intrinsic and synaptic properties of pyramidal cells in the aPC. We selectively recorded from layer II pyramidal cells which receive excitatory afferent inputs from the OB and associative cortical inputs, as well as local inhibitory inputs. To distinguish from semilunar cells, pyramidal cells were selected by somatic morphology under DIC (oval shaped vs. semilunar shaped), depth in the layer II (deeper vs. superficial), and in some cases, paired pulse ratios of evoked EPSCs were measured (facilitation vs. depression; Suzuki and Bekkers, 2011). A subset of cells with biocytin staining (*n* = 23) were constructed *post hoc* and confirmed as pyramidal cells with both apical and basal dendrites (Suzuki and Bekkers, 2011).

#### Basic Intrinsic Properties of Pyramidal Cells in Two Age Groups and NE Effects

The results comparing the intrinsic properties of P8–11 and P14+ mice were summarized in **Table 1**. Compared to the P14+ group, the P8–11 group showed higher AP threshold (–31.3 ± 1.7 mV, *n* = 16, vs. –37.7 ± 1.8 mV, *n* = 15, *t* = 2.63, *p* = 0.01), smaller AP amplitude (65.6 ± 2.2 mV, *n* = 16, vs. 77.1 ± 2.1 mV, *n* = 15, *t* = 3.72, *p* = 8.38E^-4^), wider AP half-width (1.51 ± 0.07 ms, *n* = 16, vs. 0.99 ± 0.04 ms, *n* = 15, *t* = 6.70, *p* = 2.39E^-7^), slower rising time (0.40 ± 0.02 ms, *n* = 16, vs. 0.29 ± 0.02 ms, *n* = 15, *t* = 4.19, *p* = 2.41E^-4^), and slower decay time (1.25 ± 0.08 ms, *n* = 16, vs. 0.73 ± 0.03 ms, *n* = 15, *t* = 5.91, *p* = 2.07E^-6^). Additionally, P8–11 animals exhibited more depolarized RMP than the P14+ group (–59.5 ± 1.1 mV, *n* = 22, vs. –64.3 ± 1.8 mV, *n* = 18, *t* = 2.33, *p* = 0.025). The membrane resistances within each age group did not differ (P8–11: 211.4 ± 18.8 MΩ, *n* = 23, vs. P14+: 213.6 ± 21.1 MΩ, *n* = 17, *t* = 0.075, *p* = 0.941).

**Table 1:** Pyramidal cell intrinsic properties in P8–11 and P14+ mice.

	P8–11	P14+	*t*	*P*
AP threshold (mV)	-31.3 ± 1.7	-37.7 ± 1.8	2.63	0.01^∗^
AP amplitude (mV)	65.6 ± 2.2	77.1 ± 2.1	3.72	<0.01^∗∗^
AP half width (ms)	1.51 ± 0.07	0.99 ± 0.04	6.7	<0.01^∗∗^
AP rising time (ms)	0.40 ± 0.02	0.29 ± 0.02	4.19	<0.01^∗∗^
AP decay time (ms)	1.25 ± 0.08	0.73 ± 0.03	5.91	<0.01^∗∗^
V resting (mV)	-59.5 ± 1.1	-64.3 ± 1.8	2.33	0.03^∗^
Rm (MΩ)	211.4+18.8	213.6 ± 21.1	0.08	0.94


*^∗^*p* < 0.05; ^∗∗^*p* < 0.01.*

Despite the differences in the intrinsic properties between the two age groups, NE at two concentrations (0.1 and 10 μM) had no effect in the parameters measured in either age group (**Tables 2** and **3**), except that the AP decay time showed significantly different group effects in the P14+ group with 10 μM NE [*n* = 9, *F*_(2,24)_ = 4.69, *p* = 0.02]. NE significantly reduced AP decay time (0.70 ± 0.02 ms vs. 0.76 ± 0.03 ms in control, *n* = 9, *t* = 3.05, *p* = 0.016, paired *t*-test). However, this effect was not reversed after 10 min NE washout (0.63 ± 0.04, compared to control, *t* = 3.11, *p* = 0.014).

**Table 2:** The effects of 10 μM NE on pyramidal cell intrinsic properties.

	P8-11 (*n* = 8)					P14+ (*n* = 9)				
		
	Control	NE 10μM	NE Wash	*F*_(2,21)_	*P*	Control	NE 10μM	NE Wash	*F*_(2,24)_	*P*
AP threshold (mV)	-36.1±1.5	-35.4±1.9	-36.3±2.1	0.07	0.93	-38.2±2.4	-38.4±2.4	-40.8±2.1	0.37	0.69
AP amplitude (mV)	68.1±2.9	66.8±2.3	62.9±2.5	1.11	0.35	75.7±2.8	72.9±3.3	70.5±3.7	0.63	0.54
AP half width (ms)	1.40±0.09	1.35±0.06	1.37±0.05	0.12	0.89	1.02±0.04	1.00±0.03	0.94±0.04	1.25	0.3
AP rising time (ms)	0.37±0.03	0.36±0.02	0.40±0.02	0.73	0.49	0.30±0.02	0.30±0.01	0.31±0.01	0	1
AP decay time (ms)	1.18±0.10	1.13±0.09	1.05±0.05	0.66	0.53	0.76±0.03	0.70±0.02	0.63±0.04	4.69	0.02^∗^
RMP (mV)	-63.4±3.1	-63.2±2.8	-65.2±3.7	0.12	0.89	-64.7±3.8	-63.9±4.0	-65.1±4.2	0.02	0.98
Rm (MΩ)	305.3±23.4	338.1±26.7	299.6±27.4	0.64	0.54	243.7±28.4	245.1±32.7	241.2±29.8	0	1


*^∗^p < 0.05.*

**Table 3:** The effects of 0.1 μM NE on pyramidal cell intrinsic properties.

	P8–11 (*n* = 10)					P14+ (*n* = 8)				
		
	Control	NE 0.1 μM	NE Wash	*F*_(2,27)_	*P*	Control	NE 0.1 μM	NE Wash	*F*_(2,21)_	*P*
AP threshold (mV)	-34.9±1.0	-35.4±0.8	-34.8±1.5	0.08	0.92	-36.7±1.1	-36.4±1.2	-36.4±1.5	0.02	0.98
AP amplitude (mV)	66.6±2.7	63.6±3.6	61.1±4.5	0.55	0.58	72.9±2.4	69.6±3.6	64.7±4.5	1.28	0.30
AP half Width (ms)	1.66±0.08	1.66±0.09	1.52±0.16	0.43	0.66	1.27±0.11	1.31±0.11	1.27±0.13	0.04	0.96
AP rising time (ms)	0.51±0.02	0.52±0.02	0.50±0.02	0.16	0.85	0.39±0.02	0.41±0.03	0.42±0.04	0.33	0.72
AP decay time (ms)	1.27±0.08	1.23±0.11	1.13±0.11	0.48	0.62	0.95±0.11	0.95±0.11	0.87±0.11	0.21	0.81
RMP (mV)	-60.8±1.28	-61.1±2.1	-63.9±2.16	0.79	0.46	-63.1±2.3	-63.6±2.0	-61.7±2.5	0.19	0.82
Rm (MΩ)	251.1±14.3	258.1±19.9	274.8±34.3	0.25	0.78	246.4±17.0	246.5±19.0	262.3±26.2	0.19	0.83


#### Bi-directional NE Modulations of mEPSCs in P8–11 and the Effect of β-adrenoceptor Blockade

**FIGURE 2:**
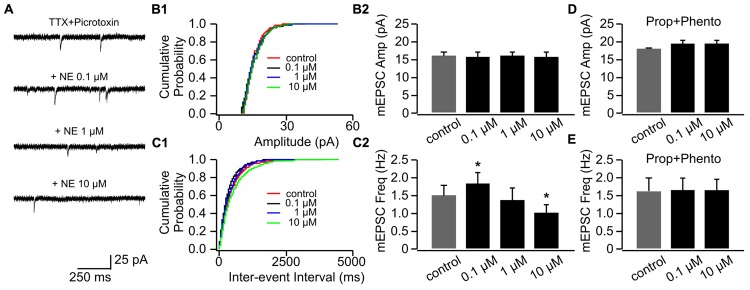
**The effects of NE on mEPSCs in P8–11.** **(A)** Example mEPSC traces of a cell with various concentrations of NE. **(B1)** Cumulative probability of mEPSC amplitudes in one cell. **(B2)** Amplitudes (Amp) of mEPSCs at various NE concentrations. **(C1)** Cumulative probability of mEPSC inter-event intervals in one cell. **(C2)** Frequencies (Freq) of mEPSCs at various NE concentrations. **(D)** Amplitudes of mEPSC at various NE concentrations in the presence of propranolol (Prop) and phentolamine (Phento). **(E)** Frequencies of mEPSCs at various NE concentrations in the presence of propranolol and phentolamine. ^∗^*p* < 0.05.

We used three doses of NE (0.1, 1, and 10 μM), which have been shown to effectively engage adrenoceptors and modify synaptic transmissions in the OB (Nai et al., 2009). **Figure 2A** shows example traces of mEPSCs recorded in one cell throughout various doses of NE. NE had no effect on mEPSC amplitudes (**Figures 2B1,B2**), however, it bi-directionally modulated mEPSC frequencies (**Figures 2C1,C2**). Low dose NE (0.1 μM) increased mEPSC frequency (1.84 ± 0.28 Hz vs. 1.51 ± 0.29 Hz in control, *n* = 7, *t* = 2.56, *p* = 0.043), whereas a high dose (10 μM) reduced mEPSC frequency (1.01 ± 0.23 Hz) compared to control (*t* = 3.48, *p* = 0.01; **Figure 2C2**). A moderate dose of NE (1 μM) had no effect on mEPSC frequency (1.38 ± 0.32 Hz, *t* = 1.09, *p* = 0.32; **Figure 2C2**). The NE effects at both low and high doses were blocked by a mixture of α- and β-adrenoceptor blockers (phentolamine 50 μM and propranolol 20 μM; **Figures 2D,E**).

**FIGURE 3:**
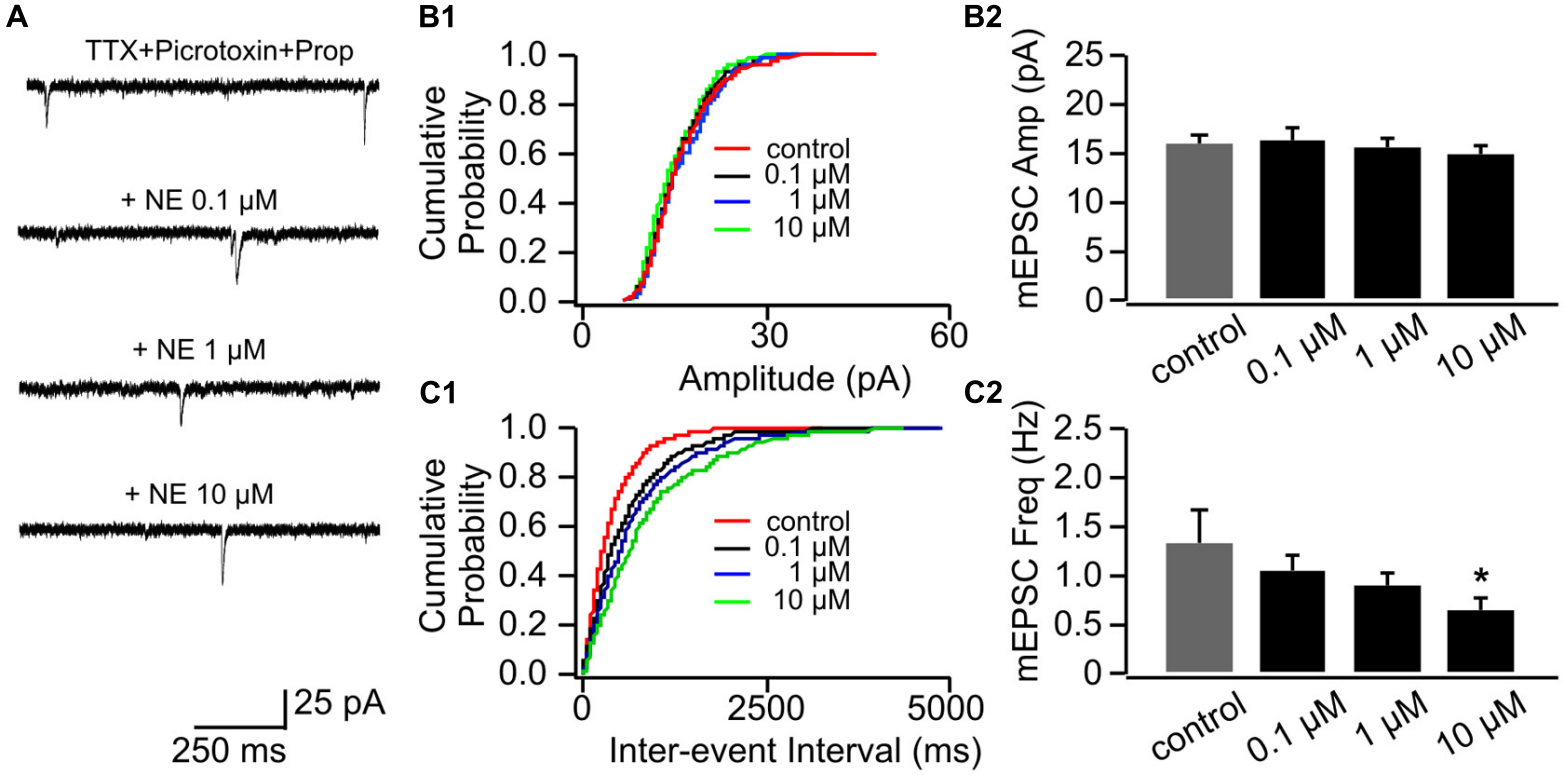
**The effects of NE in the presence of propranolol on mEPSCs in P8–11.** **(A)** Example mEPSC traces of a cell with various concentrations of NE in the presence of propranolol. **(B1)** Cumulative probability of mEPSC amplitudes in one cell. **(B2)** Amplitudes (Amp) of mEPSCs at various NE concentrations. **(C1)** Cumulative probability of mEPSC inter-event intervals in one cell. **(C2)** Frequencies (Freq) of mEPSCs at various NE concentrations. ^∗^*p* < 0.05.

Application of β-adrenoceptor antagonist propranolol had no effects on mEPSC amplitude (**Figures 3A,B1,B2**), however, it abolished the effect of 0.1 μM NE on mEPSC frequency (1.07 ± 0.16 Hz vs. 1.34 ± 0.32 Hz in control, *n* = 8, *t* = 1.43, *p* = 0.20, **Figures 3C1,C2**). Propranolol did not affect the suppressive effect of 10 μM NE on mEPSC frequency (0.67 ± 0.12) compared to control (*t* = 2.55, *p* = 0.038; **Figure 3C2**). Additionally, β-adrenoceptor blockade uncovered the inhibitory effect of NE on mEPSC frequency, since the moderate dose of NE (1 μM) reduced mEPSC frequency in the presence of propranolol (0.89 ± 0.15) compared to control although it did not reach statistical significance (*t* = 2.28, *p* = 0.055; **Figure 3C2**).

#### Suppression of mEPSCs in P14+ by NE

**FIGURE 4:**
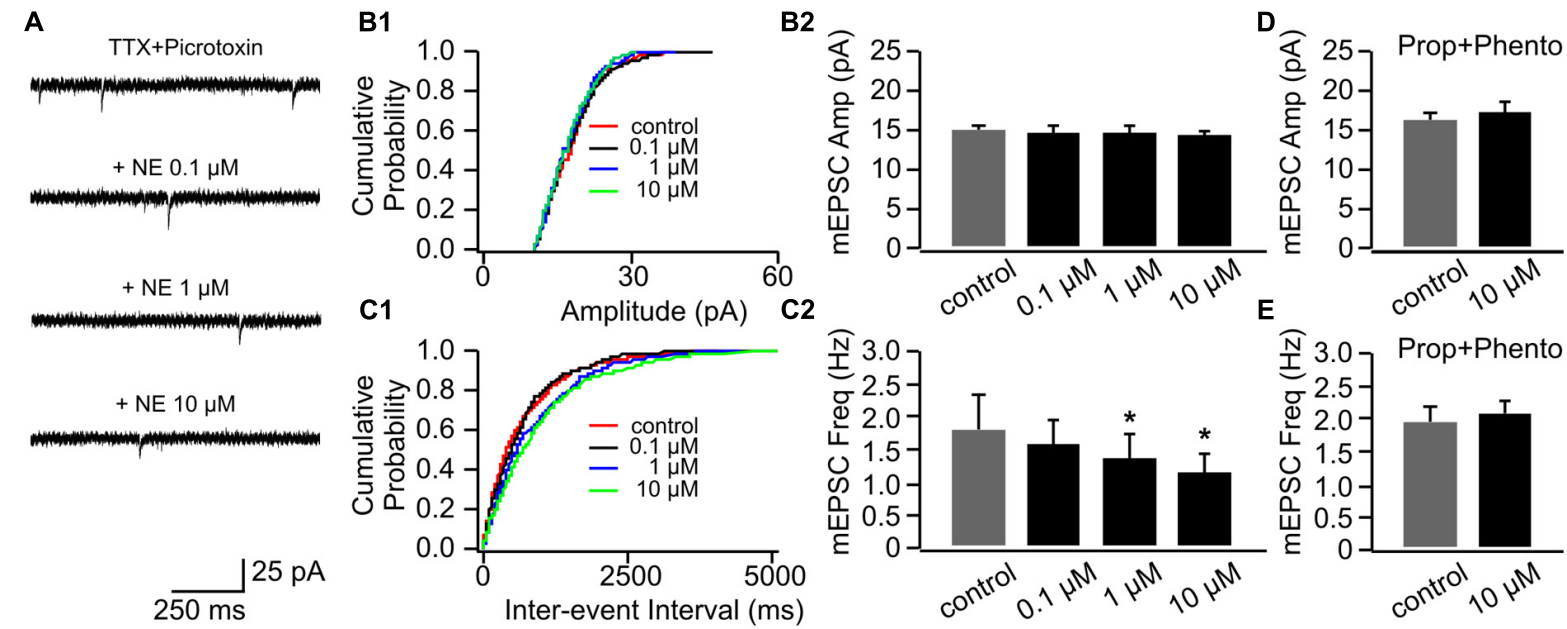
**The effects of NE on mEPSCs in P14+.** **(A)** Example mEPSC traces of a cell with various concentrations of NE. **(B1)** Cumulative probability of mEPSC amplitudes in one cell. **(B2)** Amplitudes (Amp) of mEPSCs at various NE concentrations. **(C1)** Cumulative probability of mEPSC inter-event intervals in one cell. **(C2)** Frequencies (Freq) of mEPSCs at various NE concentrations. **(D)** Amplitudes of mEPSC at various NE concentrations in the presence of propranolol (Prop) and phentolamine (Phento). **(E)** Frequencies of mEPSCs at various NE concentrations in the presence of propranolol and phentolamine. ^∗^*p* < 0.05.

NE had no effect on mEPSC amplitudes in P14+ animals (**Figures 4A,B1,B2**). However, in contrast to cells in the P8–11 group, NE at the low dose (0.1 μM) did not increase mEPSC frequency (1.5 ± 0.40 Hz vs. 1.83 ± 0.50 Hz in control, *n* = 9, *t* = 1.95, *p* = 0.087; **Figures 4C1,C2**). Furthermore, both the moderate (1 μM; 1.37 ± 0.38 Hz, *n* = 9, *t* = 3.07, *p* = 0.015) and the high dose NE (10 μM; 1.18 ± 0.26 Hz, *n* = 9, *t* = 2.54, *p* = 0.035) suppressed mEPSC frequency compared to the control (**Figure 4C2**). These suppressive effects by NE were blocked when both α- and β-blockers were added to the aCSF before NE application (**Figures 4D,E**).

Together, these results suggest α- and β-adrenoceptors have differential effects on the pyramidal cell mEPSCs in the piriform cortex. The effect of β-adrenoceptor activation is more dominate at the low dose of NE and increases presynaptic release, while α-adrenoceptors mediate inhibitory effect on presynaptic release and are predominate when NE dose is high. The lack of facilitatory effect of NE on mEPSC frequency in older pups implies that there may be developmental changes in adrenoceptor subtype expressions which alter the balance of α- and β-adrenoceptor mediated effects.

#### Bi-directional NE Modulations of mIPSCs in P8–11 and the Effect of β-adrenoceptor Blockade

**FIGURE 5:**
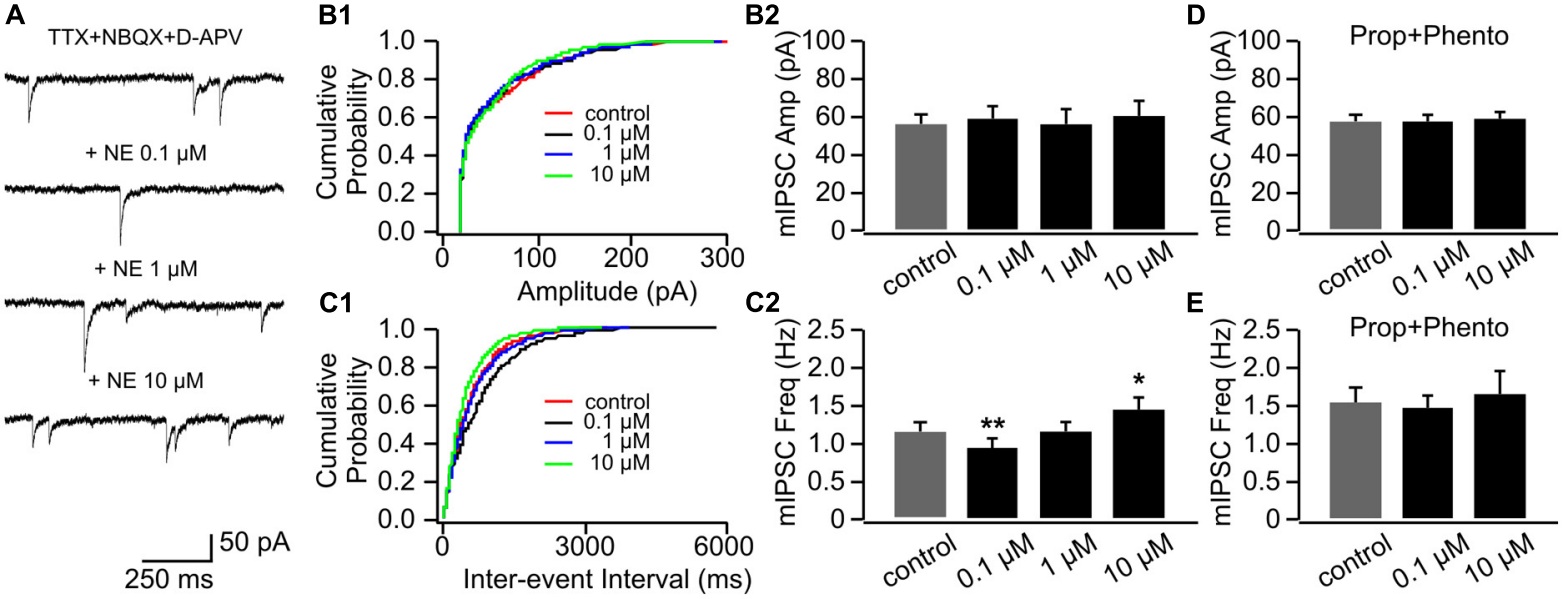
**The effects of NE on mIPSCs in P8–11.** **(A)** Example mIPSC traces of a cell with various concentrations of NE. **(B1)** Cumulative probability of mIPSC amplitudes in one cell. **(B2)** Amplitudes (Amp) of mIPSCs at various NE concentrations. **(C1)** Cumulative probability of mIPSC inter-event intervals in one cell. **(C2)** Frequencies (Freq) of mIPSCs at various NE concentrations. **(D)** Amplitudes of mIPSC at various NE concentrations in the presence of propranolol (Prop) and phentolamine (Phento). **(E)** Frequencies of mIPSCs at various NE concentrations in the presence of propranolol and phentolamine. ^∗^*p* < 0.05; ^∗∗^*p* < 0.01.

Example traces of mIPSCs with various doses of NE were shown in **Figure 5A**. NE had no effect on mIPSC amplitudes (**Figures 5B1,B2**); however, similar to its effects on mEPSCs, NE bi-directionally modulated mIPSC frequencies (**Figures 5C1,C2**). NE at the low dose (0.1 μM) significantly decreased mIPSC frequency (0.96 ± 0.09 Hz vs. 1.17 ± 0.09 Hz in control, *n* = 9, *t* = 3.87, *p* = 0.005), whereas a high dose (10 μM) increased mIPSC frequency (1.44 ± 0.15 Hz) compared to control (*t* = 2.66, *p* = 0.03; **Figure 5C2**). A moderate dose of NE (1 μM) had no effect on mIPSC frequency (1.17 ± 0.12 Hz, *t* = 0.06, *p* = 0.95; **Figure 5C2**). The NE effects at both low and high doses were blocked by a mixture of phentolamine and propranolol (**Figures 5D,E**).

**FIGURE 6:**
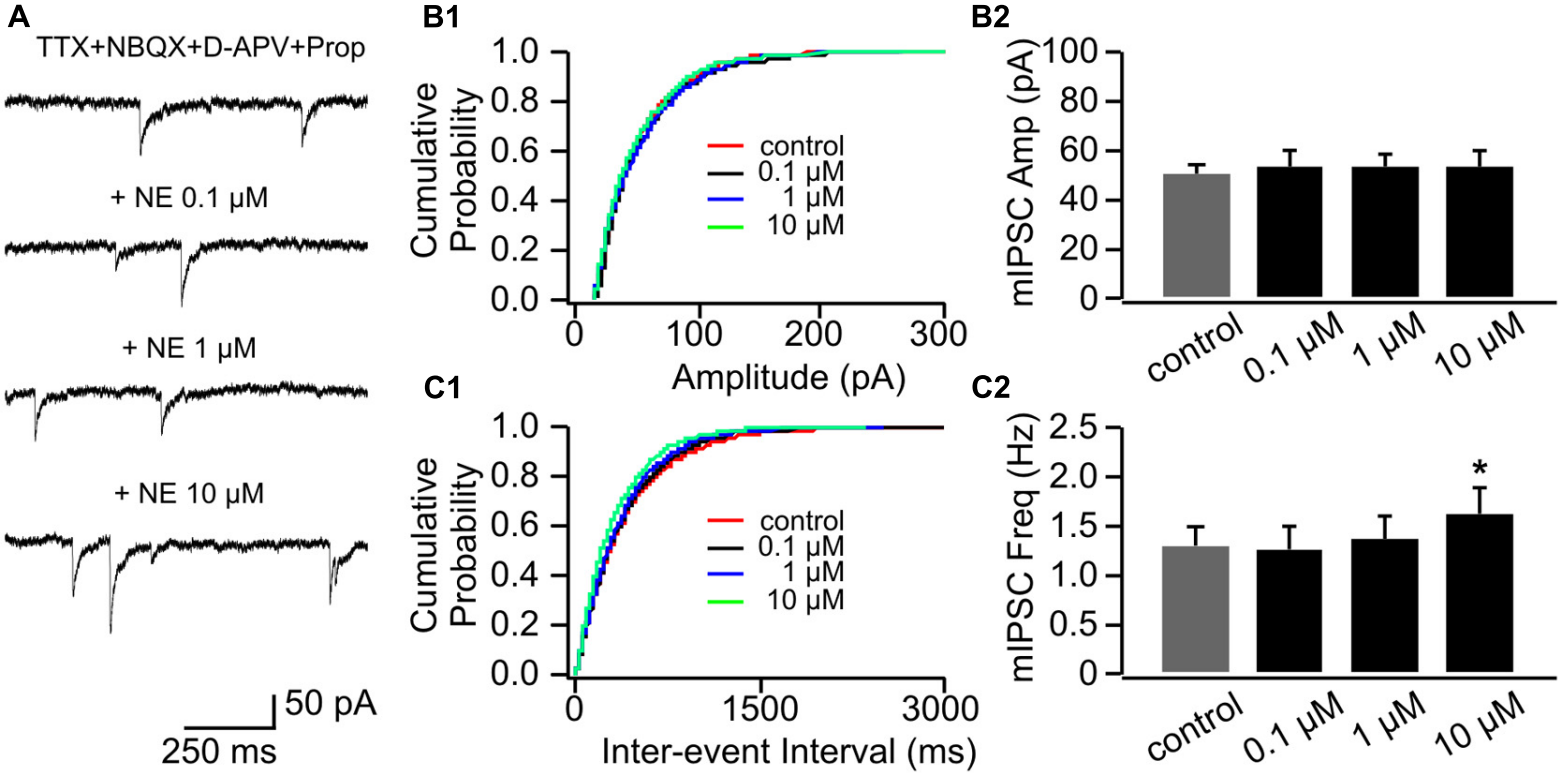
**The effects of NE in the presence of propranolol on mIPSCs in P8–11.** **(A)** Example mIPSC traces of a cell with various concentrations of NE in the presence of propranolol. **(B1)** Cumulative probability of mIPSC amplitudes in one cell. **(B2)** Amplitudes (Amp) of mIPSCs at various NE concentrations. **(C1)** Cumulative probability of mIPSC inter-event intervals in one cell. **(C2)** Frequencies (Freq) of mIPSCs at various NE concentrations. ^∗^*p* < 0.05.

Application of propranolol itself had no effect on mIPSC amplitude (**Figures 6A,B1,B2**), however, the effect of 0.1 μM NE on mIPSC frequency was abolished in the presence of propranolol (1.26 ± 0.24 Hz vs. 1.28 ± 0.22 Hz in control, *n* = 8, *t* = 0.21, *p* = 0.84, **Figures 6C1,C2**). Propranolol did not alter the effect of 10 μM NE on mIPSC frequency (1.62 ± 0.26) compared to control (*t* = 3.28, *p* = 0.014; **Figure 6C2**). Moderate dose of NE (1 μM) did not affect mIPSC frequency significantly in the presence of propranolol (1.38 ± 0.24) compared to control (*t* = 1.60, *p* = 0.153; **Figure 6C2**).

#### Enhancement of mIPSCs in P14+ by NE

**FIGURE 7:**
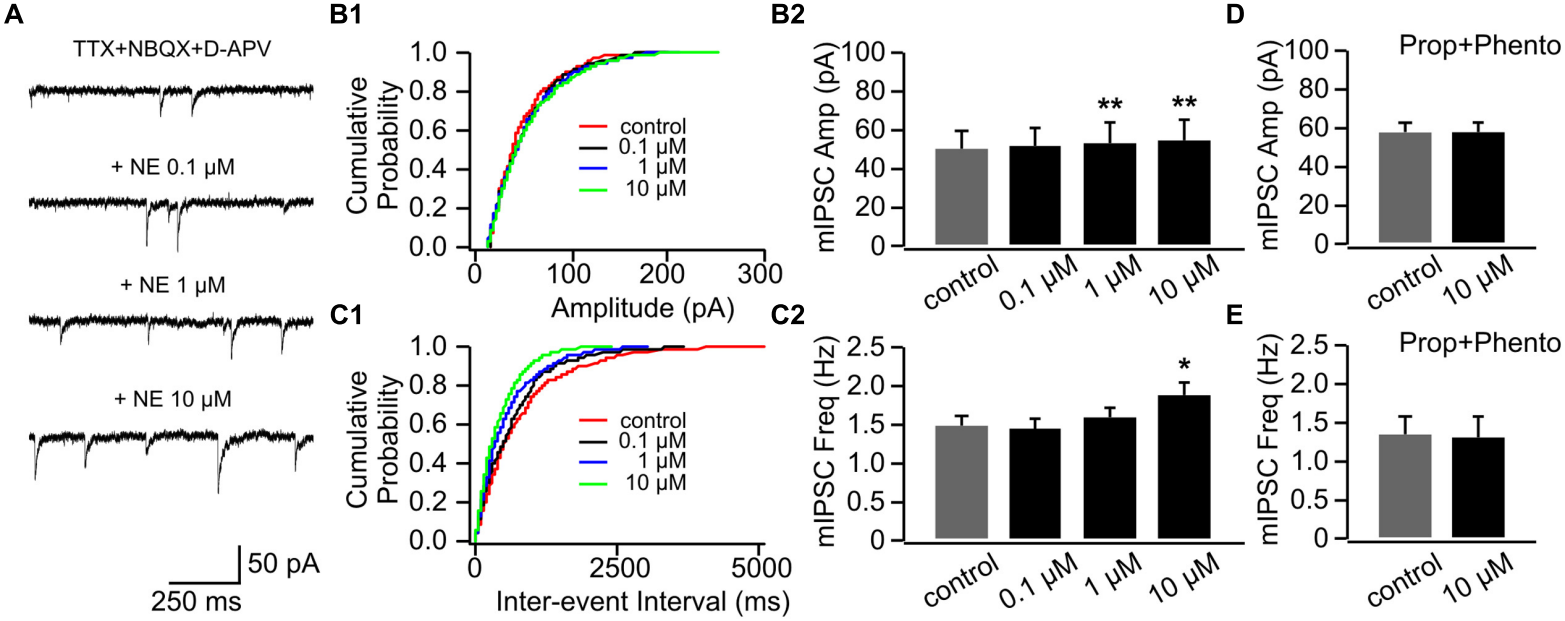
**The effects of NE on mIPSCs in P14+.** **(A)** Example mIPSC traces of a cell with various concentrations of NE. **(B1)** Cumulative probability of mIPSC amplitudes in one cell. **(B2)** Amplitudes (Amp) of mIPSCs at various NE concentrations. **(C1)** Cumulative probability of mIPSC inter-event intervals in one cell. **(C2)** Frequencies (Freq) of mIPSCs at various NE concentrations. **(D)** Amplitudes of mIPSC at various NE concentrations in the presence of propranolol (Prop) and phentolamine (Phento). **(E)** Frequencies of mIPSCs at various NE concentrations in the presence of propranolol and phentolamine. ^∗^*p* < 0.05; ^∗∗^*p* < 0.01.

Interestingly, NE consistently increased mIPSC amplitudes in P14+ animals with the higher doses (1 and 10 μM; **Figures 7A,B1,B2**). The mIPSC amplitude increased to 53.98 ± 10.08 pA with 1 μM NE from 50.59 ± 9.53 pA in control condition (*n* = 7, *t* = 4.18, *p* = 0.006) and further increased to 55.55 ± 10.24 pA with 10 μM NE (*t* = 5.24, *p* = 0.002; **Figures 7A,B1,B2**). However, in contrast to cells in the P8–11 group, NE at the low dose (0.1 μM) did not decrease mIPSC frequency (1.45 ± 0.13 Hz vs. 1.48 ± 0.15 Hz in control, *n* = 7, *t* = 0.26, *p* = 0.802; **Figures 7C1,C2**). The high dose NE (10 μM; 1.89 ± 0.13 Hz) increased mIPSC frequency compared to the control (*t* = 2.76, *p* = 0.033; **Figure 7C2**). The effects of NE on both mIPSC amplitude and frequency were blocked when both α- and β-adrenoceptor blockers were used in the presence of NE (**Figures 7D,E**). Consistent with mEPSC recording, β-adrenoceptors appear to be activated by lower concentration of NE compared to α-adrenoceptors at inhibitory synapses and β-adrenoceptor effect (lowering mIPSC frequency) is more dominate in younger mouse pups.

## Discussion

In this work we established β-adrenoceptor mediated early odor preference learning in mice. Odor preference learning was seen at P8, but not P14. We characterized the effects of NE in aPC layer II pyramidal cells in two age groups (P8–11 and P14+). Pyramidal cells in aPC undergo developmental changes in their intrinsic electrophysiological properties (RMP and APs) from P8 to weaning age (P21). Two different doses of NE did not have clear effects on intrinsic properties in either age group. However, NE did differentially modulate synaptic properties in two age groups in a dose dependent manner. Low dose (0.1 μM) of NE promoted excitation of the pyramidal cells only in P8–11 pups by increasing mEPSC frequency and by decreasing mIPSC frequency *via* β-adrenoceptors. However, in a higher dose (10 μM), NE facilitated inhibition by decreasing mEPSCs and increasing mIPSCs in both age groups.

### Age-dependent Intrinsic Properties of Pyramidal Cells and Adrenergic Modulations

In both the somatosensory (Lorenzon and Foehring, 1993; Maravall et al., 2004) and visual cortex (Kasper et al., 1994a,b), AP properties and membrane resistance in pyramidal cells change rapidly during the postnatal weeks. Pyramidal cells in layer 5 of the visual cortex are not fully mature until postnatal 3–4 weeks (Kasper et al., 1994b). The RMP becomes more negative and input resistance decreases with age. The AP configurations change during this postnatal period (Kasper et al., 1994b). In rat barrel cortex, less spike adaptation in mature neurons is related to the strength of slow afterhyperpolarization (AHP) and coincides with the closure of the critical period for sensory map formation (Maravall et al., 2004). We show here, for the first time, that intrinsic properties of pyramidal cells in the olfactory cortex undergo similar postnatal development. More depolarized RMP and AP threshold along with wider APs were observed in P8–11 pups compared to P14+.

Interestingly, NE (two doses) did not affect the majority of the intrinsic properties in either age group, with the exception of a decreased AP decay time in P14+ mice in the presence of 10 μM NE. NE modulation of AP configuration has been shown in other systems by either increasing potassium conductance (Slack, 1986) or decreasing calcium conductance (Dunlap and Fischbach, 1978). Both α- and β-adrenoceptors are expressed in layer II pyramidal cells in the PC (Wanaka et al., 1989; Nicholas et al., 1993a,b; Pieribone et al., 1994). Lack of NE effect on other intrinsic properties can possibly be explained by opposing actions of adrenoceptor subtypes co-existing on the same cells (Bai and Renaud, 1998; Carette, 1999), or existence of mixed subpopulations of pyramidal cells expressing differential levels of adrenoceptor subtypes. In the adult rat PC, NE also state-dependently alters neuronal excitability by regulating AHP channels (Brosh et al., 2006). AHP modulation affects spiking patterns responding to sensory input. It would be interesting in the future to determine whether such modulations exist in mouse pups and if they exhibit age-dependent changes.

### Age-dependent Adrenergic Modulations of Pyramidal Cell Synaptic Properties

In rats, NE release via LC stimulation enhances sensory processing in the PC by strengthening odor-evoked pyramidal cell responses and sharpening signal patterns (Bouret and Sara, 2002). NE bi-directionally modulates synaptic transmission in the rat olfactory cortex slice (Collins et al., 1984). Low concentration of NE (0.1–5 μM) facilitates transmission at the lateral olfactory tract to pyramidal cell synapses, while high dose (20–250 μM) depresses transmission (Collins et al., 1984). In another study, high dose NE (≥10 μM) was shown to cause stronger suppression of associational synaptic transmission in layer Ib (Hasselmo et al., 1997), which would result in enhanced signal to noise ratio at afferent synapse from the OB (in layer Ia; Linster and Hasselmo, 2001). However, the precise synaptic sites and actions of NE on pyramidal cells were not clear. Here we demonstrate age-dependent modulations of NE on pyramidal cell synaptic currents. Low dose NE is excitatory in P8–11 mouse pups. NE at 0.1 μM concentration increased mEPSC frequency and decreased mIPSC frequency, suggesting that both effects are presynaptic in nature. Blockade of β-adrenoceptor abolished both NE effects on mEPSC and mIPSC. However, in the older pups (P14+), NE at the low dose had no effects on either mEPSC or mIPSC. In contrast, high dose NE is inhibitory in both age groups. NE at 10 μM suppressed mEPSC and enhanced mIPSC. While presynaptic actions accounted for NE effects in P8–11 mice, modulation of both pre- and post-synaptic sites were involved in inhibitory synapses in P14+ mice. The effects by high dose NE, at least in younger mice (P8–11), were not affected by β-adrenoceptor blockade. Together, these results suggest that α- and β-adrenoceptors mediate the inhibitory and the excitatory effects of NE, respectively, consistent with opposing actions of these receptor subtypes reported in other brain areas (Kobayashi, 2007; Salgado et al., 2012). In both the aPC (Marek and Aghajanian, 1996), and OB (Nai et al., 2010), α_1_-adrenoceptor mediates excitation of GABAergic interneurons by NE. In the OB, α_1_-adrenoceptor is activated with higher dose of NE than α_2_- or β-adrenoceptors (Nai et al., 2009). The developmental changes in NE actions are likely attributed to increased α_1_- and/or decreased β-adrenoceptor expression/function with age. Future recordings with specific adrenoceptor agonists in two age groups will address the developmental changes in the ratio of α- and β-adrenoceptor actions.

Consistent with our findings, β-adrenoceptor activation *in vivo* with isoproterenol reverses a short-term depression mediated by presynaptic mGluRs (Best and Wilson, 2004). Isoproterenol could counter presynaptic depression by activating cAMP-dependent protein kinase, which phosphorylates mGluRs and inhibits their function (Best and Wilson, 2004). Consistently, isoproterenol increases presynaptic release and LTP at the lateral olfactory tract to pyramidal cell synapses in neonatal rat slices (Morrison et al., 2013). How β-adrenoceptor mediates the depression of presynaptic release at the inhibitory synapse in P8–11 mice is less clear. Interestingly, opposing effects of β_1_- and β_2_-adrenoceptors on synaptic transmission have been observed in layer V/VI pyramidal cells of the rat prefrontal cortex (Ji et al., 2008; Zhou et al., 2013; Luo et al., 2014). A presynaptic PKA-dependent pathway was shown to be involved in β_1_-adrenoceptor induced suppression of glutamate release in the prefrontal cortex (Luo et al., 2014). Thus, increased release at the excitatory synapse and decreased release at the inhibitory synapse by low dose NE in P8–11 animals are likely mediated by different β-adrenoceptor subtypes or through different molecular signaling.

### NE Modulation in the aPC and Sensitive Period Odor Preference Learning

Early evidence suggested that initial odor memory is encoded in the OB. Sullivan et al. (2000b) showed that LC-induced NE release in the OB is both necessary and sufficient for early odor preference learning. NE via multiple-adrenoceptor activations in the OB enhances mitral cell excitation (Hayar et al., 2001; Yuan, 2009; Shakhawat et al., 2012), facilitates gamma oscillation in the bulbar network (Gire and Schoppa, 2008) and promotes a stronger and more synchronized output to the olfactory cortex (Yuan et al., 2014).

More recent studies support aPC as another critical player in the network. Early odor preference learning results in enhanced c-fos (Raineki et al., 2009) and *Arc* (Shakhawat et al., 2014) expressions in aPC pyramidal cells in response to the conditioning odor. Transient silencing of the aPC during training prevented the expression of an odor preference memory acquired with a fully functional OB (Morrison et al., 2013). Like the OB, β-adrenoceptor activation in the aPC is both necessary and sufficient for early odor preference learning (Morrison et al., 2013). Pairing activation of aPC β-adrenoceptors using isoproterenol with peppermint odor produces a significant preference for peppermint 24 h later. This effect is blocked by co-infusion of the β-adrenoceptor blocker propranolol. Propranolol infusion alone in the aPC prevents odor preference learning induced by pairing odor with stroking, suggesting both stages of olfactory processing (OB and aPC) require β-adrenoceptor activation in order to support preference memory.

Here we show a β-adrenoceptor mediated early odor preference learning also exists in mice. We propose that a β-adrenoceptor mediated excitatory effect of NE in young animals may underlie the heightened plasticity for learning during the sensitive period. Increased presynaptic release at glutamatergic synapses and reduced release at GABAergic interneuron synapses promote NMDAR-dependent LTP of pyramidal cells and associative learning. Future tests of NE on evoked synaptic responses and pyramidal cell spiking properties will provide more compelling evidence.

In older mice, α-adrenoceptor mediated inhibitory effects appear to dominate. Increased inhibition of pyramidal cells by NE coincides with reduced plasticity and termination of the sensitive period learning. In older animals, tactile stimulation ceases to promote LC NE release (Kimura and Nakamura, 1987). However, NE was shown to be critically involved in the specificity of odor habituation memory (Mandairon et al., 2008) and discrimination of difficult odor pairs (Doucette et al., 2007; Escanilla et al., 2010). NE is released due to emotional arousal and attention in these cases; however, the local concentration of synaptically released NE in the PC is unknown. Increased inhibition by NE may sharpen the signal to noise ratio and promote pattern separation during odor discrimination in adult rodents (Linster et al., 2011).

## Author Contributions

QY and XC designed the experiments. AG, NP, and QY conducted the experiments and analysis. AG, NP, and QY wrote the paper.

## Conflict of Interest Statement

The authors declare that the research was conducted in the absence of any commercial or financial relationships that could be construed as a potential conflict of interest.
